# Stenting for Symptomatic Intracranial Vertebrobasilar Artery Stenosis in Northeast of China: A Single-Center Study

**DOI:** 10.3389/fneur.2020.609286

**Published:** 2021-02-16

**Authors:** Zhongxiu Wang, Chao Wang, Chao Li, Mingchao Shi, Shouchun Wang, Yi Yang

**Affiliations:** ^1^Department of Neurology, Stroke Center & Clinical Trial and Research Center for Stroke, The First Hospital of Jilin University, Changchun, China; ^2^China National Comprehensive Stroke Center, Changchun, China; ^3^Jilin Provincial Key Laboratory of Cerebrovascular Disease, Changchun, China

**Keywords:** symptomatic intracranial vertebrobasilar artery stenosis, stenting, outcome, complication, safety

## Abstract

**Objective:** We described the incidence of surgery-related complications to evaluate the safety of endovascular therapy for severe symptomatic intracranial vertebral basilar artery stenosis (IVBS) in our stroke center in Northeast of China.

**Methods:** Consecutive patients with symptomatic IVBS caused by 70–99% stenosis despite standard medical treatment of antiplatelet agents plus statin were enrolled. Either balloon-mounted stent or balloon predilation plus self-expanding stent was performed. Clinical adverse events such as stroke, transient ischemic attack (TIA), and death after the surgery were documented. Radiological events such as in-stent thrombosis, dissection, and guide-wire perforation during the process were recorded as complications as well. The baseline characteristics and outcomes of patients among different Mori types were compared.

**Results:** From January 2017 to December 2018, 97 patients with stroke or TIA due to intracranial IVBS were treated by stenting, including 30 patients with basilar artery (BA) stenosis, 55 patients with intracranial vertebral artery (V4) stenosis, and 12 patients with V4-BA stenosis. The primary events include two intracranial hemorrhage (2.1%, 2/97), seven ischemic events (7.2%, 7/97), and two death (2.1%, 2/97). The successful stent deployment rate was 98.9% (96/97). The Apollo stents were used more for Mori A lesions. Self-expanding stents were more used in Mori C lesions. Mori C lesions were more vulnerable to endovascular procedure and showed higher rate of complications than A (*p* = 0.008) and B type (*p* = 0.047).

**Conclusion:** A high technical success rate of IVBS stenting could be achieved, and the safety was acceptable, whereas Mori C lesions were more vulnerable to endovascular procedure and showed a higher rate of complications than A and B types.

## Introduction

Posterior circulation stroke takes ~25% of all ischemic stroke cases, and unlike anterior stroke, symptomatic vertebrobasilar atherosclerotic disease is more challenging and has a relatively higher annual recurrence of stroke despite standard medical treatment of antiplatelet agents and statin (1–3), and the symptoms caused by large artery stenosis or occlusion are often devastating. Also, it was associated with a risk of stroke > 20%, as was shown in a pooled data analysis from two studies (4, 5). Stenting is becoming a promising therapeutic method for recurrent ischemic events refractory to best medical treatment, but there are also side effects such as procedure-related ischemic stroke or transient ischemic attack (TIA), intracranial hemorrhage, and even death (6, 7). Former researches such as the Stenting and Aggressive Medical Management for Preventing Recurrent Stroke (SAMMPRIS) study (8) and the Vitesse Intracranial Stent Study for Ischemic Therapy (VISSIT) trial (9) used stents, either self-expanding or balloon-expandable ones, and demonstrated higher perioperative stroke and death rate compared with medical therapy, showing less favorable outcome of this procedure. However, not only advanced devices have emerged, but also more experienced hands have grown, making this technique more applicable and favorable (10). Because of different anatomy and function, the posterior circulation arteries might have different characteristics compared with that of anterior circulation for endovascular treatment (1). Moreover, intracranial artery stenosis is more frequently seen in Asian population than in white and black patients (11), and the northeast area of China holds people with one of the highest rates of stroke in the world, whose profiles have rarely been published. Thus, we aimed to investigate the safety of stenting for patients with severe intracranial vertebral BA stenosis (IVBS) refractory to medical treatment.

## Methods

### Participants

This study was approved by the ethics committee of the First Hospital of Jilin University. Written informed consent was obtained from all patients and/or their relatives. Patients' baseline characteristics and outcomes were collected. Inclusion criteria for endovascular stenting were as follows (5): (1) 18 to 85 years old; (2) vertebral artery V4 segment or basilar artery atherosclerotic stenosis 70% or greater as defined by the Warfarin Aspirin Symptomatic Intracranial Disease Trial criteria (12) and a lesion length of ≤ 15 mm on digital subtraction angiography (DSA), with normal distal vessel; (3) recent non-disabling stroke or TIA of the vertebrobasilar vascular territory within 90 days refractory to standard medical therapy (stroke or TIA recurrence due to severe stenosis of VA and basilar artery (BA) under strict control of risk factors such as hypertension and diabetes mellitus [DM] and more than 3 months' use of at least one antiplatelet drug and statin, or stroke or TIA recurrence due to hypoperfusion < 3 months even under aggressive medical treatment: dual antiplatelet drugs and control of low-density lipoprotein cholesterol <1.8 mmol/L or >50% decrease); (4) a modified Rankin score (mRS) ≤ 3; (5) at least one atherosclerotic risk factor (hypertension, DM, hyperlipidemia, and cigarette smoking); and (6) hypoplastic posterior communicating artery (PcomA) and/or posterior cerebral artery P1 segment, and for V4 segment lesion, the contralateral vertebral artery should be hypoplastic or occluded. Exclusion criteria included (1) non-atherosclerotic stenosis; (2) patients with stroke symptoms that were not thromboembolic or hemodynamic (including perforator strokes); (3) intracranial hemorrhage in the territory of the stenotic artery within 6 weeks; (4) potential source of cardiac embolism; (5) concurrent intracranial tumor, aneurysm, and cerebral arteriovenous malformation; (6) tandem ≥50% stenosis of extracranial carotid or vertebral artery; (7) known contraindication to heparin, aspirin, clopidogrel, anesthesia, and contrast media; (8) platelet count <100,000; (9) international normalized ratio >1.5 (irreversible) and uncorrectable bleeding diathesis; (10) and life expectancy <1 year because of other medical conditions.

We use Mori classification of intracranial artery stenosis to differentiate lesion morphology (13): type A (<5 mm in length, concentric or moderately eccentric lesions not totally occlusive), type B (tubular, 5–10 mm in length, extremely eccentric or totally occluded lesions), and type C (10–15 mm in length, extremely angulated lesions with excessive tortuosity of the proximal segment, or totally occluded lesions). The medical treatment for risk factor control was based on the SAMMPRIS study and the Chinese ischemic stroke guideline. Clinical and radiological adverse events such as stroke, TIA, death, in-stent thrombosis, dissection, and guide-wire perforation were recorded as complications. Technical success was defined angiographically as <30% residual stenosis (5). TIA was defined as a reversible neurological deficit that lasts for at least 10 min and completely resolved within 24 h regardless of the diffusion-weighted imaging changes.

### Outcome Measures

The primary outcome was any stroke (including ischemic or hemorrhagic), TIA, and death caused by the endovascular procedure during hospital stay. The secondary outcome was successful revascularization (residual stenosis <30%) rate, 90-day mRS of patients with complications after stenting. Other radiological events, including in-stent thrombosis and dissection, were also recorded as adverse events. If a new posterior circulation stroke was suspected, computed tomography (CT) or magnetic resonance imaging scans were arranged for documentation.

### Follow-Up

Radiological and clinical follow-up information of patients with complications after surgery was obtained during in hospital. Ninety-day clinical outcomes were reviewed and collected by a trained neurologist who was blinded to treatment via face-to-face or telephone interview.

### Statistical Analysis

Statistics analysis was performed with the SPSS version 16.0 (SPSS, Chicago, IL, USA). The baseline, imaging, and stenting data of all patients are presented as means (± SD) or median interquartile range for continuous variables and number for categorical data. Continuous variables were tested with the Student *t*-test, whereas categorical data were tested with χ^2^ test or Fisher exact test (when the expected cell frequency was <5). When continuous variables had skewed distributions, the Mann–Whitney *U*-test was used to identify the difference in the continuous variables. The significant *P*-value was set at < 0.05.

## Results

From January 2017 to December 2018, 97 patients (67 males, aged 64.4 ± 8.6) with stroke or TIA due to IVBS were treated by stenting, including 30 patients with BA stenosis, 55 patients with V4 stenosis, and 12 patients with V4-BA stenosis. Vertebrobasilar artery stenosis and poor collaterals were all confirmed by DSA before stenting.

The successful stent deployment rate was 98.9% (96/97). Taking all the other adverse events into account, the rate of overall complication was 22.7% (22/97). And the primary events include two cases of intracranial hemorrhage (2.1%, 2/97): one was caused by wire penetration of BA during the surgery and the other one was due to intimal damage confirmed by postoperative CT scan. Seven ischemic events happened within 24 h after the surgery including one TIA and six strokes (7.2%, 7/97), all caused by perforator injury: two happened in V4 group and five happened in BA group. Two patients died (2.1%, 2/97) during hospital stay, one was caused by subarachnoid hemorrhage due to wire penetration, and the other one was caused by medulla oblongata infarction due to perforator injury. Other complications included eight (8.2%, 8/97) patients with thrombosis during the procedure process, three (3.1%, 3/97) patients with dissection after balloon dilation, and two (2.1%, 2/97) patients with hyperperfusion. Among all the 22 patients, nine (40.9%) showed deterioration by increasing at least one National Institutes of Health Stroke Scale (NIHSS) score during in-hospital stay, whereas 17 patients (77.3%) recovered to independence (mRS <3) (Table 1) in 90 days' follow-up.

**Table 1:** Complication profile and prognosis.

**No**.	**Gender**	**Age**	**Meri type**	**Target artery**	**Intervention method**	**Complication**	**Remedial measure**	**Pre-NIHSS**	**Post-NIHSS**	**New symptoms after stenting**	**90d mRS**
1	M	60	B	V4-BA	Ballon mounted stent	Perforator injury	Drug therapy	0	0	Numbness of left extremities	0
2	M	68	B	V4	Ballon + balloon mounted stent	Perforator injury	Drug therapy	2	2	Nystagmus, nausea	0
3	M	47	B	V4	Ballon mounted stent	Thrombosis	IA urokinase	0	0	None	0
4	M	52	A	V4	Ballon + self-expending stent	Hemorrhage	BP control	0	0	Mild headache	0
5	M	70	B	V4	Ballon + self-expending stent	Perforator injury	Drug therapy	2	2	Mild weakness of right limbs	0
6	F	71	B	V4	Ballon + self-expending stent	Perforator injury	Drug therapy	0	0	Stroke recurrence 2 months later	3
7	M	54	A	V4	Ballon + self-expending stent	Perforator injury	Drug therapy	0	35	Coma	6
8	F	61	C	BA	Fail	Hemorrhage	Protamine + BP control	2	25	Paralysis, disturbance of consciousness, dyspnea	6
9	M	55	B	V4	Ballon + self-expending stent	Thrombosis	IA urokinase	0	0	Dizziness, nausea	1
10	M	53	A	V4	Ballon + self-expending stent	Thrombosis	IA urokinase	0	0	Transient dysarthria	0
11	M	49	B	BA	Ballon + self-expending stent	Perforator injury	Drug therapy	2	15	Right limbs paralysis	5
12	M	61	C	BA	Ballon + self-expending stent	Dissection	Stenting	1	9	Right limbs paralysis	5
13	M	66	A	V4	Ballon + self-expending stent	Perforator injury	Drug therapy	2	3	Dysarthria, dizziness	2
14	M	44	B	V4	Balloon mounted stent	Thrombosis	Mechanical thrombectomy	0	3	Ataxia, nystagmus	2
15	M	59	A	BA	Ballon + self-expending stent	Dissection	Stenting	0	0	None	0
16	F	54	B	BA	Ballon + self-expending stent	Hyper-perfusion	BP control	1	1	Headache, restlessness	0
17	F	65	A	V4	Ballon + self-expending stent	Hyper-perfusion	BP control	2	2	Headache, nausea	1
18	M	67	B	BA	Ballon + self-expending stent	Thrombosis	IA tirofiban	0	5	Left limb and facial paralysis	1
19	M	59	A	V4	Ballon + self-expending stent	Thrombosis	IA tirofiban + urokinase	4	6	Right limbs paralysis	3
20	M	43	B	V4-BA	Ballon + self-expending stent	Dissection	Stenting	1	1	Transient paralysis of left limbs	2
21	M	49	A	V4	Ballon + self-expending stent	Thrombosis	IA urokinase	0	1	Transient nystagmus, nausea, dysarthria	1
22	M	53	A	V4	Ballon + self-expending stent	Thrombosis	IA tirofiban + urokinase	3	3	None	0

The baseline characteristics of patients with or without surgery-related complications are presented in Table 2. We found no statistical differences on rate of hypertension, DM, coronary heart disease, atrial fibrillation, prior ischemic stroke, smoking, and alcohol drinking between these two groups.

**Table 2:** Clinical characteristics and complications.

**Characteristic**		**Complication**		***P***
		**Yes** ***n* = 22**	**No** ***n* = 75**	
Age	y ± SD	57.3 ± 8.3	58.9 ± 8.5	0.423
Male sex	%(n)	72.7 (16)	68.0 (51)	0.673
Coronary heart disease	%(n)	22.7 (5)	16.0 (12)	0.329
Atrial fibrillation	%(n)	13.6 (3)	10.7 (8)	0.708
Cerebral infarction/TIA	%(n)	18.2 (4)	22.7 (17)	0.451
Hypertension	%(n)	81.8 (18)	61.3 (46)	0.075
Diabetes mellitus	%(n)	45.5 (10)	26.7 (20)	0.08
Alcohol drinking	%(n)	50.0 (11)	59.2 (45)	0.404
Cigarette smoking	%(n)	59.0 (13)	48.0 (36)	0.421
BA	n	6	24	0.508
V4	n	14	41	0.716
V4-BA	n	2	10	1
Balloon-mounted stent	n	3	20	0.53
Balloon + balloon-mounted stent	n	1	5	1
Balloon + self-expanding stent	n	17	49	0.086
Mori A	n	5	33	0.538
Mori B	n	7	29	0.047
Mori C	n	10	13	0.008^*^
Lesion length	mm ± SD	8.9 ± 4.16	6.8 ± 3.4	0.141
Stenosis percentage	% ± SD	86.4 ± 7.69	84.7 ± 8.21	0.787

* *P < 0.05 for comparing between two groups*.

As for whether the site of the stent would influence the rate of complication, we found no difference among patients who had stenting for BA (6/30, *p* = 0.508), V4 (14/55, *p* = 0.716), or V4-BA (2/12, *p* = 1). Nor did we find statistical difference of interventional techniques (balloon-mounted stent or balloon+ balloon-mounted stent or balloon+ self-expanding stent) in these segments of arteries on complication occurrence (3/23 vs. 1/6 vs. 17/66; *p* = 0.53 vs. 1.0 vs. 0.086).

While Mori C lesions were more vulnerable to endovascular procedure and showed higher rate of complications than A type (*p* = 0.008) and B type (*p* = 0.047). Tables 3, 4 show that as for BA and Mori C type lesion stenting, self-expanding stent such as Wingspan or Enterprise were more frequently applied (*p* = 0.029, *p* = 0.018). Compared to types B and C lesions, Mori A and V4 lesions were more likely to receive balloon-mounted stent (*p* = 0.006).

**Table 3:** Lesion distribution and stenting method.

**Lesion location**	**Self-expanding stent**	**Balloon-mounted stent**	***P*-value**
BA	25	4	0.029^*^
V4	35	20	0.843
V4-BA	8	4	0.202

* *P <0.05 for comparing between two groups*.

**Table 4:** Lesion type and stenting method.

**Lesion type**	**Self-expanding stent**	**Balloon-mounted stent**	***P*-value**
A	20	18	0.006^*^
B	29	6	0.980
C	19	4	0.018^*^

* *P <0.05 for comparing between two groups*.

## Discussion

The rate of overall complications in our study was 22.7% (22/97), which was higher than that reported in literature (14), because we included both radiological and clinical abnormalities during and after the procedure. Patients with radiological complications may not necessarily present clinical symptoms, but these abnormalities could compromise the integrity of the vessel wall or interfere the blood flow especially when occurring on the opening of an artery (15, 16). Thus, we took into account all these radiological changes as complications even if they did not necessarily show clinical symptoms as to present the characteristics of a lesion. For instance, we found type C lesions were more vulnerable to endovascular procedure and showed a higher rate of these complications, which means higher risks of symptom deterioration or new infarction that also have a clinical meaning: It is a reminder that we could not be too cautious when dealing with it.

Previous studies have shown that stenting for IVBS was challenging because of its higher complication rate and severe symptoms once it happened. Levy et al. (17) treated 11 IVBS patients; three suffered periprocedural deaths and one died of pontine stroke. Tsang et al. (18) conducted a systematic review and random-effects meta-analysis of all available randomized controlled trials evaluating the safety and efficacy of percutaneous transluminal angioplasty and stenting (PTAS), in comparison with medical therapy, for symptomatic intracranial artery stenosis (sICAS) and found a higher risk of any stroke or death within 2 years in the sICAS subgroup located in posterior circulation than medical treatment. SAMMPRIS (8) and VISSIT trial (9) both demonstrated higher perioperative stroke and death rate compared with medical therapy, showing less favorable outcome of this procedure. It was obvious that stenting for IVBS was no easy thing, and complications could occur; none of these results recommended stenting as first-line treatment for IVBS, but with the advancement of neurointervention devices and more experienced neurointerventionists, safety of PTAS for IVBS seemed to be acceptable. Miao et al. (19) treated 159 intracranial atherosclerotic disease (ICAD) patients with balloon-mounted stent and 141 ICAD patients with balloon plus self-expanding stent. The 30-day rate of stroke, TIA, and death was 4.3%. Ding et al. (15) analyzed 19 ICAD stenting series after SAMMPRIS trial, including 2,196 patients with 2,314 lesions, showing the median rate of postprocedural ischemic events was 9.4% (range, 0–25%). Thus, we believe that after a thorough clinical assessment and exquisite surgery procedure, a particular group of patients would benefit from IVBS stenting with high successful rate of stent deployment and low rate of complication occurrence such as stroke or death.

We collected and compared clinical data of patients with or without any kind of complications in order to distinguish patients who might suffer from surgery-related complications. As for medical history, we found no statistical difference of these factors between groups, but patients with hypertension and DM seemed to have a higher occurrence of complications, which explains that these patients have higher rates of perforator stroke and poor collateral status.

The practice that perforator stroke was more likely to happen after BA stenting was also reported in previous literatures (19). We assumed that compared with intracranial vertebral arteries, the BA had much more perforating branches and poor collateral circulation, which made it extremely sensitive to ischemia. Also, neurological deficits caused by the occlusion of perforator orifice of BA were severe and sometimes even lethal. This may be caused by atherosclerotic debris being displaced over the perforator origins during angioplasty or stent deployment. The SAMMPRIS trial concluded that the occlusion of perforating arteries was the most common cause of ischemic stroke especially after BA PTAS.

The rate of successful stent deployment in our center was 98.9% (96/97), and device selection of self-expandable stent or balloon-mounted stent depended on arterial access and lesion morphology (7, 20). From our experience, the Gateway–Wingspan system with its excellent flexibility in traversing curvatures is more suitable for tortuous lesions, whereas the Apollo stent is preferred for patients with smooth arterial access, which does not require exchanging. Therefore, for patients with smooth arterial access and Mori A lesion, the balloon-mounted stent would be convenient. For patients with tortuous arterial access and a Mori B or C lesion, Gateway balloon plus self-expandable stents such as Wingspan stent system is preferred. And if perforator arteries originated near the stenotic site, predilation of small-sized balloon plus self-expandable stent is also preferred out of protection for the orifice of the perforator artery, as we believed that balloon-mounted stent might hurt the perforator arteries more easily, which could cause devastating result. Following these easy strategies while doing the endovascular procedure, we did not find difference of complication rate among patients who had stenting for BA, V4, or V4-BA. Nor did we find any difference of interventional techniques (balloon-mounted stent or balloon + self-expanding stent) in these segments of artery. But we did have seven ischemic events happened, including one TIA and six strokes, all caused by perforator injury. Among all the 22 patients, nine (40.9%) showed NIHSS deterioration by increasing at least 1 point. Two patients died (2.1%, 2/97): one was caused by subarachnoid hemorrhage due to wire penetration, and the other one was caused by medulla oblongata infarction due to perforator injury, whereas 17 patients (77.3%) recovered to independence (mRS <3). Patients may show symptoms such as dizziness, nausea, and nystagmus, and mostly reached recession very soon. Our study showed even IVBS was challenging because of its higher complication rate; the majority could recovery to independency.

Some limitations should be considered when interpreting the findings because this is a single-center observational study with a relatively small size of patients. And we analyzed only the clinical data we collected during the hospital stay; thus, the long-term prognosis of this treating strategy such as stroke recurrence or restenosis is still to be investigated. Nevertheless, we described our experience and findings of stenting for IVBS in the population of northeast China.

## Conclusions

A high technical success rate of IVBS stenting could be achieved, and the safety was acceptable, whereas Mori C lesions were more vulnerable to endovascular procedure and showed higher rate of complications than A and B types.

## Data Availability Statement

The raw data supporting the conclusions of this article will be made available by the authors, without undue reservation.

## Ethics Statement

The studies involving human participants were reviewed and approved by Human and Research Ethics committees of the First Hospital of Jilin University. The patients/participants provided their written informed consent to participate in this study.

## Author Contributions

ZW and CW conceived and designed the study, acquired the data, and drafted and revised the manuscript. All authors analyzed and interpreted the data and critically revised the manuscript for important intellectual content.

## Conflict of Interest

The authors declare that the research was conducted in the absence of any commercial or financial relationships that could be construed as a potential conflict of interest.
